# Chromosome number alterations cause apoptosis and cellular hypertrophy in induced pluripotent stem cell models of embryonic epiblast cells

**DOI:** 10.1242/bio.061814

**Published:** 2025-01-24

**Authors:** Althea Stella Anil Martis, Loshini Soundararajan, Pallavi Shetty, Syed Moin, Tejashree Vanje, Yogeshwaran Jai Sankar, Shagufta Parveen

**Affiliations:** Manipal Institute of Regenerative Medicine, Manipal Academy of Higher Education, Manipal 576104, India

**Keywords:** Chromosomal aneuploidies, Abortive embryonic development, Epiblast model, Apoptosis, Micronuclei, Hypertrophy

## Abstract

Chromosomal aneuploidies are a major cause of developmental failure and pregnancy loss. To investigate the possible consequences of aneuploidy on early embryonic development *in vitro*, we focused on primed pluripotent stem cells that are relatable to the epiblast of post-implantation embryos *in vivo*. We used human induced pluripotent stem cells (iPSCs) as an epiblast model and altered chromosome numbers by treating with reversine, a small-molecule inhibitor of monopolar spindle 1 kinase (MSP1) that inactivates the spindle assembly checkpoint, which has been strongly implicated in chromosome mis-segregation and aneuploidy generation. Upon reversine treatment, we obtained cells with varied chromosomal content that retained pluripotency and potential to differentiate into cells of three germ lineages. However, these cells displayed lagging chromosomes, increased micronuclei content, high *p53* expression and excessive apoptotic activity. Cell proliferation was not affected. Prolonged *in vitro* culture of these cells resulted in a selective pool of cells with supernumerary chromosomes, which exhibited cellular hypertrophy, enlarged nuclei, and overproduction of total RNAs and proteins. We conclude that increased DNA damage responses, apoptosis, and improper cellular mass and functions are possible mechanisms that contribute to abnormal epiblast development.

## Teaser

**Summary:** Reversine-treated human induced pluripotent stem cells as models of aneuploid epiblast suggest apoptosis and cellular hypertrophy caused by imbalanced gene dosages due to chromosome number variations as possible mechanisms of abortive embryonic development.

## INTRODUCTION

Aneuploidy is an incorrect number of chromosomes in cells. Aneuploidy of embryonic cells is a leading cause of pregnancy failures (Gruhn et al., 2019; Essers et al., 2023; Rana et al., 2023; Sun et al., 2023; Takenouchi et al., 2024; Zhai et al., 2024). Embryonic aneuploidies arise during oocyte meiotic divisions (Charalambous et al., 2023; Essers et al., 2023; Takenouchi et al., 2024) or zygotic mitotic divisions (Vanneste et al., 2009; McCoy et al., 2023). Inherent cellular mechanisms that cause chromosome mis-segregation include relaxed cell cycle control checkpoints, defective chromosome cohesion, aberrations of centrosome and mitotic spindle, and DNA replication stress (Schneider and Ellenberg, 2019; Dumont et al., 2020; Palmerola et al., 2022; Mihajlović et al., 2023; Mihalas et al., 2024; Zhang and Zheng, 2024). Aneuploidy of human embryos causes implantation failure, developmental failure, congenital defects with subsequent pregnancy losses and miscarriages. All monosomies except for X chromosome and most trisomies cause embryonic lethality in humans (Santaguida et al., 2017). Aneuploid cells within embryos are either progressively depleted by apoptosis or spatiotemporally allocated to extraembryonic organs (Lebedev, 2011; Orvieto et al., 2020; Yang et al., 2021). Thus, only embryos with sufficient chromosomal integrity survive to term, while those with excessive and unresolvable aneuploid cells result in pregnancy wastage (Essers et al., 2023).

The pluripotent epiblast cells constitute a fundamental phase of the post-implantation embryo. It is the epiblast cells that gastrulate and develop into the embryo proper (Wang et al., 2024). Though it is established that presence of aneuploid cells has an overall deleterious effect on embryonic development, the altered cellular behaviour and outcomes of aneuploid epiblast cells are incompletely understood.

To this end, we used induced pluripotent stem cells (iPSCs) as *in vitro* models of epiblast cells (Li et al., 2012; Weinberger et al., 2016; Zhang et al., 2016; Neagu et al., 2020; Wang et al., 2021; Bi et al., 2022). We altered chromosome numbers in iPSCs by impairing spindle assembly checkpoint (SAC) activity with reversine, an inhibitor of monopolar spindle 1 kinase (MSP1), the kinase that initiates the SAC signalling cascade. Reversine is known to cause severe chromosome segregation defects that efficiently generate aneuploidy in many cell types (Bolton et al., 2016; Santaguida et al., 2017). After standardising the dose and duration of reversine treatment on iPSCs, we studied the consequences of chromosome number variations at a cellular level using this model. We observed irregular karyotypes, increased apoptosis, presence of micronuclei and lagging chromosomes shortly after reversine treatment.

When the treated iPSCs were continued in culture for recovery, we found selective survival and enrichment of polysomic cells versus monosomic cells, implying the tolerance of polyploidy and death of pluripotent cells with chromosomal monosomy. Monosomy is a viably unstable and a karyotypically less-fit trait that results in cells being unable to propagate. The polyploid cells showed hypertrophy with higher total RNA and protein content corresponding to chromosome dosage. With these findings, we propose that post-chromosome mis-segregation cells with monosomies are eliminated by apoptosis while cells with polyploidies survive *in vitro*. However, the surviving cells have an altered cellular mass that interferes negatively with proper development, possibly causing foetal malformations and death.

## RESULTS

### Treatment of pluripotent stem cells with reversine

**Fig. 1.**
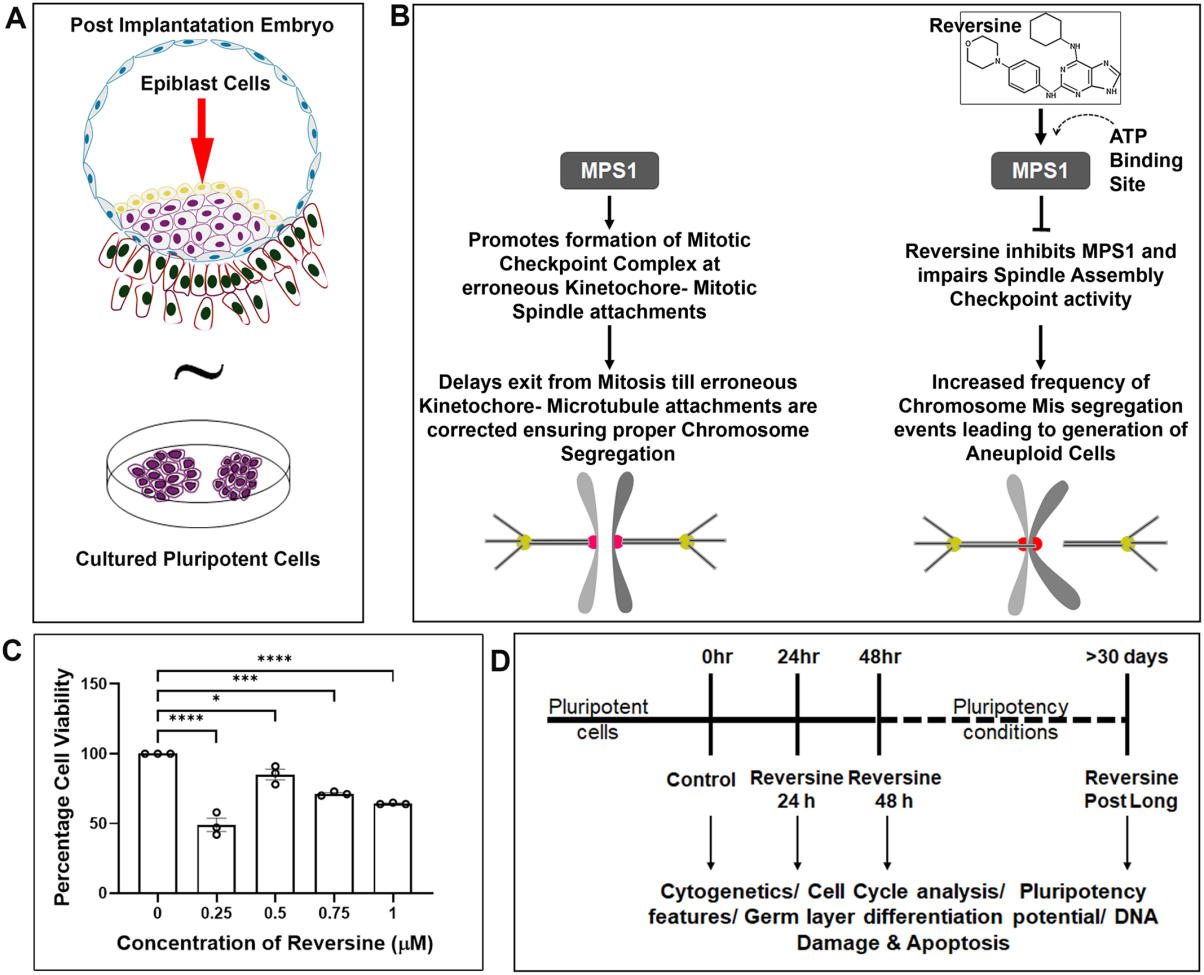
**Treatment of pluripotent stem cells with reversine.** (A) Primed pluripotent stem cells as a model of human embryonic epiblast cells. (B) Reversine is a 2, 6 disubstitute purine that is an ATP-competitive inhibitor of human MPS1. Illustration of inhibition of MPS1 by reversine, impairing activity of the mitotic checkpoint complex, causing chromosomal number alterations. (C) Determination of optimal concentration of reversine for the study using MTT assay. *n*=3 experimental repeats; one-way ANOVA. *P<0.05, ***P<0.001, ****P<0.0001. Error bars denote mean±s.e.m. (D) Study design.

We chose iPSCs as an elementary model of post-implantation epiblast cells (Fig. 1A). SAC components ensure the proper segregation of chromosomes during mitosis. Perturbation of SAC activity will cause chromosomal instability, resulting in alterations in chromosome copy numbers. To induce chromosome mis-segregation in iPSCs we used reversine, a potent inhibitor of MPS1, a protein kinase (Santaguida et al., 2010) (Fig. 1B). 3-(4,5-Dimethylthiazol-2-yl)-2,5-diphenyltetrazolium bromide (MTT) assay was performed to assess cell viability while determining the reversine dose. Based on treatments at which the cell viability was highest, a dose of 0.5 µM was chosen in the experimental design (Fig. 1C). iPSCs were treated with reversine for 24 and 48 h durations, hereafter called reversine 24 h and reversine 48 h, respectively. Reversine was withdrawn post 48-h treatment, and cells were maintained in prolonged culture to check for recovery and resulting phenotypes, hereafter called reversine post long. Cells from each condition were removed for further experiments (Fig. 1D).

### Reversine induces chromosome mis-segregation in pluripotent cells and disrupts the cell cycle

Several methods have been devised to experimentally induce chromosome mis-segregation in cells. Compounds that interfere with microtubule dynamics or microtubule-kinetochore attachment induce chromosome mis-segregation, albeit with mitotic arrests through SAC activation. To avoid such cell cycle arrest caused by a delay mitosis exit, we decided to generate cells with mis-segregated chromosomes by perturbing SAC function rather than activating the checkpoint. SAC inactivation does not arrest cells in mitosis; it instead hastens progression through mitosis even when chromosomes are not correctly attached to the spindle fibres, resulting in aneuploid progeny cells (Santaguida et al., 2017).

**Fig. 2.**
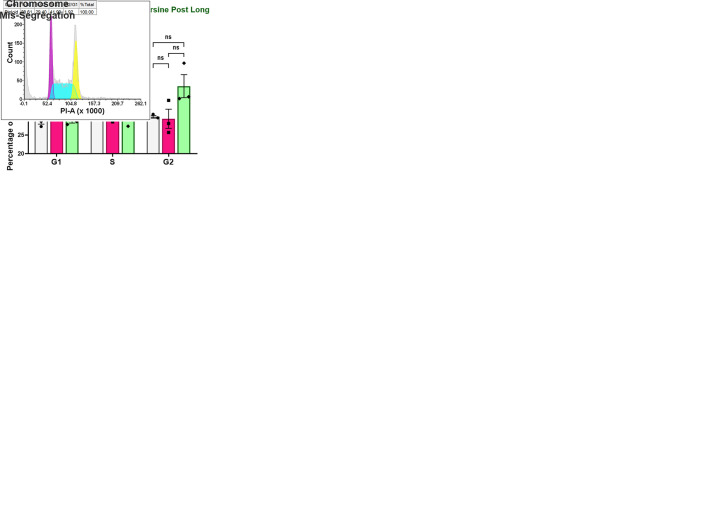
**Reversine induces chromosome mis-segregation in pluripotent cells and disrupts the cell cycle.** (A) Schematic of experimental setup. Control, reversine 24 h and reversine post long cells were subjected to karyotyping and flow cytometry-based DNA content analysis (representative karyotype images of reversine-treated cells). (B) Chromosome counts from metaphase spreads of control, reversine 24 h and reversine post long cells. (C) Representative cell cycle plots depicting disruption of cell cycling upon treatment with reversine. (D) Flow cytometry-based quantification of G2/M phase arrest. (E) Quantification of cells in G1, S and G2/M phases of the cell cycle in control, reversine 24 h and reversine post long cells induced by exposure to reversine (0.5 µM). *n*=3 experimental repeats; paired two-tailed *t*-test. *P<0.05; ns, *P*>0.05. Error bars denote mean±s.e.m.

We used karyotyping and propidium iodide staining-flow cytometry workflow to detect the chromosomal content of reversine-treated iPSCs (Fig. 2A). As expected, reversine treatment resulted in chromosome mis-segregation in iPSCs. Giemsa staining was performed to detect chromosome content in the iPSCs after treatment with reversine. While control iPSCs had a normal count of 46 chromosomes, reversine-treated cells were karyotypically heterogeneous, with random chromosome losses and gains. Reversine 24 h cells had metaphase spreads, with chromosome numbers ranging from less than 40 to greater than 50. Reversine 48 h cells could not be karyotyped due to excessive cell death during the experimental treatment, making it difficult to obtain spreads for Giemsa staining and further processing. Reversine post long cells had chromosome numbers mostly greater than 50 (Fig. 2B,C).

The overall chromosomal content was also determined by propidium iodide staining and flow cytometry analysis of DNA content. The control cells showed conventional cell cycling with 2N-containing G1 cells and 4N-containing G2/M cells, along with a proportion of cells in the S phase. G1 and G2/M phases had normally distributed Gaussian peaks of PI fluorescence. The G2/M peak in the histogram had a mean position twice that of the G1 peak. The S phase had a typical Gaussian-broadened distribution with early and late S-phase peaks overlapping with G1 and G2/M. The DNA content histogram of reversine 24 h cells was skewed, with broader G1, S and G2/M peaks. The mean positions of G1 and G2/M peaks had shifted and occupied fluorescence ranges outwardly flanking the fluorescence peaks of control cells.

This suggested the presence of cells with less than 2N and more than 4N DNA content, confirming mis-segregated chromosomes in cells. In the DNA content histogram of reversine post long cells, the mean position of G1 peak was twice that of G2/M of control cells, denoting that these cells had a chromosomal content of approximately 4N and post replication had a DNA content of more than 8N (Fig. 2D). Twenty-four hours of reversine treatment significantly increased the percentage of cells in G1 phase while decreasing the percentages of cells in the S and G2/M phases (Fig. 2E). Taken together, the karyotyping and cell cycle data suggest that reversine treatment causes chromosome mis-segregation in iPSCs effectively. After confirming altered chromosome numbers in iPSCs, we moved on to analysing the consequences of the altered chromosomal content at a cellular level in the iPSCs.

### Reversine-treated cells retain pluripotency and the ability to form embryoid bodies

**Fig. 3.**
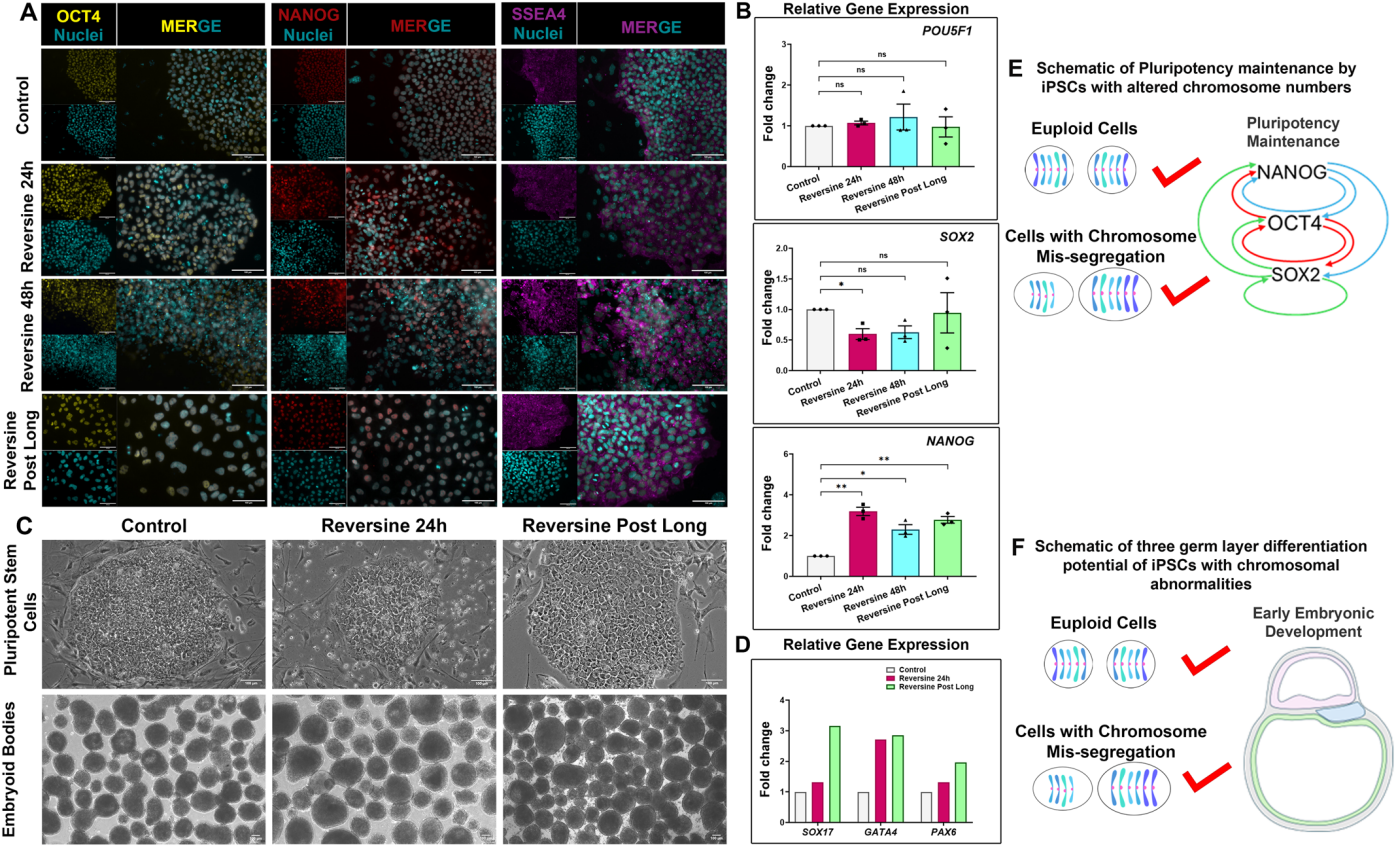
**Reversine-treated cells retain pluripotency and the ability to form embryoid bodies.** (A) Validation of pluripotency by immunocytochemistry. Representative images of control and treated iPSCs stained for OCT4, NANOG and SSEA4, with positively stained cells indicating retention of pluripotency upon reversine treatment. Scale bars: 100 μm. The images were processed in FIJI software (http://imagej.nih.gov/ij/). (B) qPCR analysis for pluripotency gene expression showing that expression of *POU5F1*, *SOX2* and *NANOG* was comparable between control and treated iPSCs. Gene expression normalised to *ACTB*. *n*=3 experimental repeats; paired two-tailed *t*-test. *P<0.05, **P<0.01; ns, *P*>0.05. Error bars denote mean±s.e.m. (C) Representative phase-contrast micrographs of control and reversine treated-iPSC colonies. Reversine 24 h colonies have small gaps between cells. Reversine post long colonies have larger sized cells. Lower row shows embryoid bodies generated with control and treated iPSCs. The embryoids show no apparent irregularities. Scale bars: 100 μm. (D) qPCR data of gene expression of three germ layer transcripts – *SOX17*, *GATA4* and *PAX6* – in embryoid bodies. Gene expression normalised to *ACTB*. *n*=1 experimental repeat. (E,F) Schematic representations of results of this section.

To ascertain that the obtained aneuploid iPSCs retained pluripotency, immunocytochemistry for pluripotency transcription factors OCT4 and NANOG, along with the cell surface glycolipid SSEA4, was performed. The cells stained positive for the pluripotency markers with no apparent reduction in fluorescent intensities in comparison to the control iPSCs (Fig. 3A). However, the distribution of cells in colonies of reversine 24 h and reversine 48 h iPSCs was less compact compared to that in control colony cells, possibly owing to cell death. The colony compactness was restored upon prolonged culture as observed in reversine post long iPSCs. The nuclei were enlarged in reversine 24 h and 48 h iPSCs typical of aneuploid cells (Yang et al., 2021). The nuclear size, as well as cell size, had dramatically increased in the reversine post long cells. This could be accounted for by the presence of supernumerary chromosomes present in the nuclei and a relatable overproduction of proteins in these cells causing hypertrophy (Fig. 3A,C). Quantitative PCR (qPCR) analysis of pluripotency-related expression of *POU5F1* (*OCT4*), *SOX2* and *NANOG* genes was also performed to confirm maintenance of pluripotency post reversine treatment (Fig. 3B).

There was no significant reduction in expression of *POU5F1* and *SOX 2*, but increased expression of *NANOG*, observed in the reversine-treated iPSCs. This could be explained by the fact that reversine is a MEK1/2 inhibitor (Chen et al., 2007, 2024) and inhibition of MEK1/2 stabilises *NANOG* (Mulas et al., 2024), thereby increasing *NANOG* expression in these iPSCs.

Next, we generated embryoid bodies to examine the potential of obtained chromosomally abnormal cells to initiate differentiation into the three germ layers of endoderm, mesoderm and ectoderm typical of early gastrulation. All groups of iPSCs were able to form compact embryoid bodies upon aggregation. The embryoid bodies expressed genes of endoderm (*SOX17*), mesoderm (*GATA4*) and ectoderm (*PAX6*). These results verify that the chromosomally altered iPSCs retain the potential to differentiate into three germ layers relatable to early gastrulation. Thereby, chromosome mis-segregation did not alter pluripotency traits or the capacity to initiate differentiation to three germ layers (Fig. 3C,D). Schematic representations of results of this section can be found in Fig. 3E,F.

### Pluripotent cells show signs of DNA damage and increased apoptosis post reversine treatment, which recovers after prolonged culture *in vitro*

Next, we moved on to study the effects of aneuploidy on pluripotent cellular physiology. During the first 24 h of reversine treatment, negligible cell death was observed in the cultures, followed by significant cell death between 36 and 48 h in the form of cell floaters. Upon removal of reversine after 48 h, relatively high cell death continued for an additional 5-6 days. Gradually, cell death decreased with recovery of iPSCs accompanied by cell proliferation and steady re-establishment of compact colonies in cultures. These iPSCs could be conveniently maintained by conventional pluripotent stem cell culture methods and were propagated in prolonged culture for over 60 days. The above karyotyping and cell culture results suggest that, during the first few cycles of cell division, chromosome mis-segregation occurred, with chromosomes being unequally distributed in two daughter cells during mitosis. A random pool of daughter cells with monosomic and polysomic chromosomal numbers were generated. During subsequent cell divisions, the cells with monosomic chromosomes underwent cell death, perhaps due to haploid insufficiencies and below threshold levels of gene dosage required to meet cellular demands. The polysomic cells maintain viability, albeit with high gene-dosage imbalances.

Lagging chromosomes move slower than other chromosomes during anaphase, causing chromosome mis-segregation and subsequently leading to micronuclei formation (Hosea et al., 2024). Micronuclei are small membrane-bound cytoplasmic structures composed of whole or fragmented chromosomes that are excluded from the primary nucleus. The presence of micronuclei is a mark of DNA segregation errors (Klaasen et al., 2022; Truong et al., 2023; Takaki et al., 2024).

**Fig. 4.**
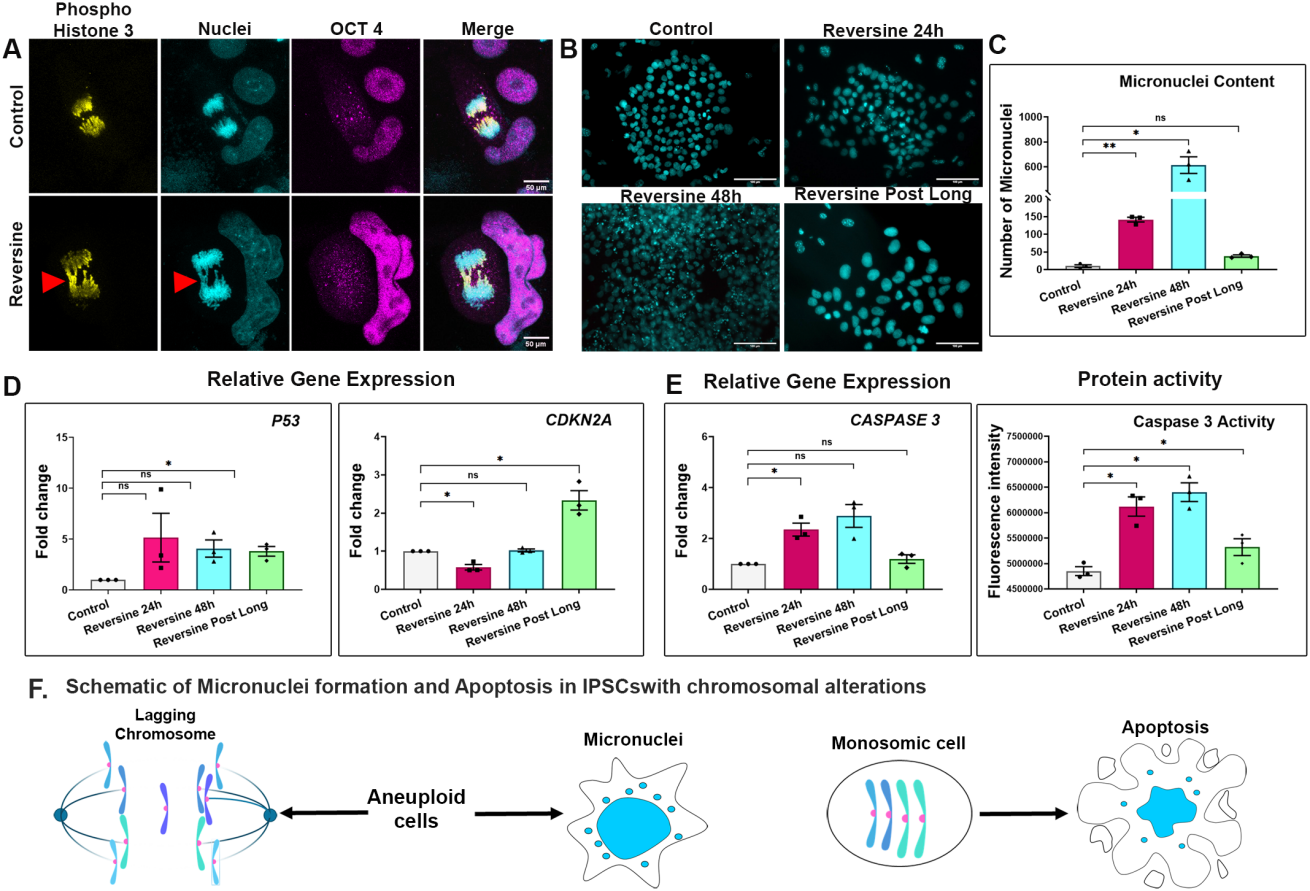
**Pluripotent cells show signs of DNA damage and increased apoptosis post reversine treatment, which recovers after prolonged culture *in vitro*.** (A) Representative images of phospho-histone 3-positive mitotic cells showing the presence of lagging chromosomes upon treatment with reversine. Scale bars: 50 μm. (B) Prevalence of micronuclei in cells treated with reversine compared to control cells shown by DAPI staining. Scale bars: 100 μm. (C) Quantification of micronuclei by DAPI staining. The images were processed in FIJI software (http://imagej.nih.gov/ij/). (D) Relative gene expression quantification of DNA damage marker *p53* and cell cycle inhibitor *CDKN2A* by qPCR. (E) Gene and protein expression quantification of apoptosis marker caspase 3 by qPCR and fluorometric enzyme activity. (F) Schematic representation of results of this section. Gene expression normalised to *ACTB*. *n*=3 experimental repeats; paired two-tailed *t*-test. *P<0.05, **P<0.01; ns, *P*>0.05. Error bars denote mean±s.e.m.

To examine if chromosome mis-segregation is causing any additional DNA damage in cells, we conducted microscopic examination of iPSCs stained with the nuclear dye DAPI, phospho-histone H3 to mark mitotic cells and OCT4 to mark pluripotent cells. The reversine-treated iPSCs harboured lagging chromosomes evident in anaphase, indicating improper microtubule-kinetochore attachments in those cells (Fig. 4A). DAPI staining also revealed the presence of numerous micronuclei in the reversine 24 h and reversine 48 h cells. The quantity of micronuclei increased with duration of treatment, with reversine 48 h cells having nearly four times more micronuclei than that of reversine 24 h cells. Reversine post long cells had a negligible micronuclei content, indicating recovery and lack of persisting DNA damage (Fig. 4B,C).

Next, we checked if the reversine-induced aneuploidy had upregulated the p53 pathway as a response to DNA damage. Indeed, p53 gene expression was upregulated nearly 4-fold in reversine 24 h and reversine 48 h cells. High expression of p53 gene was also found in reversine post long cells despite the absence of micronuclei (Fig. 4D). We assume that is due to increased gene dosage in these polyploid cells. The p53 pathway, in turn, activates several downstream effector processes including cell cycle arrest and apoptosis (Aubrey et al., 2018; Abuetabh et al., 2022). To this end, we analysed the levels of a cell cycle biomarker, *CDKN2A*, and apoptosis biomarker caspase 3. *CDKN2A* encodes two alternatively spliced transcripts: *p16INK4A*, an inhibitor of CDKs 4/6, and *p14ARF*, an indirect activator of p53 (Stott et al., 1998). Gene expression analysis revealed downregulation of *CDKN2A* in reversine-treated cells, suggesting non-inhibition of cell cycle in these cells (Fig. 4D). Lack of cell cycle arrest was also confirmed by the fact that treatment-surviving iPSCs could maintain rapid proliferation in culture. These observations are in agreement with previous studies reporting lack of p53-dependent cell cycle arrest as well as downregulated Ink4/Arf locus encoded by *CDKN2A* in pluripotent stem cells (Li et al., 2009; Menendez et al., 2010; He et al., 2016; Jaiswal et al., 2020).

We continued the study by analysing the extent of apoptosis by examining the levels of caspase 3 in the cells. We observed a 2- and 2.5-fold increase in levels of caspase 3 gene expression in reversine 24 h and reversine 48 h cells, respectively. A relevant and concomitant increase of caspase 3 protein activity was also recorded. Caspase 3 gene expression and protein activity were notably reduced to near control levels in reversine post long cells, implying that these cells were not undergoing apoptosis despite harbouring polyploid number of chromosomes (Fig. 4E).

With the above results, we conclude that pluripotent cells bypassed cell cycle arrest due to DNA damage. Cells harbouring monoploidies were eliminated by apoptosis, probably due to their inability to sustain required gene doses for survival. Cells with supernumerary chromosomes outlasted the experimental induction of chromosomal mis-segregation and could propagate in culture albeit with higher gene dosages. A schematic representation of the results of this section can be found in Fig. 4F.

### The surviving iPSCs harbouring supernumerary chromosomes have increased cell size with higher cellular RNA and protein content

**Fig. 5.**
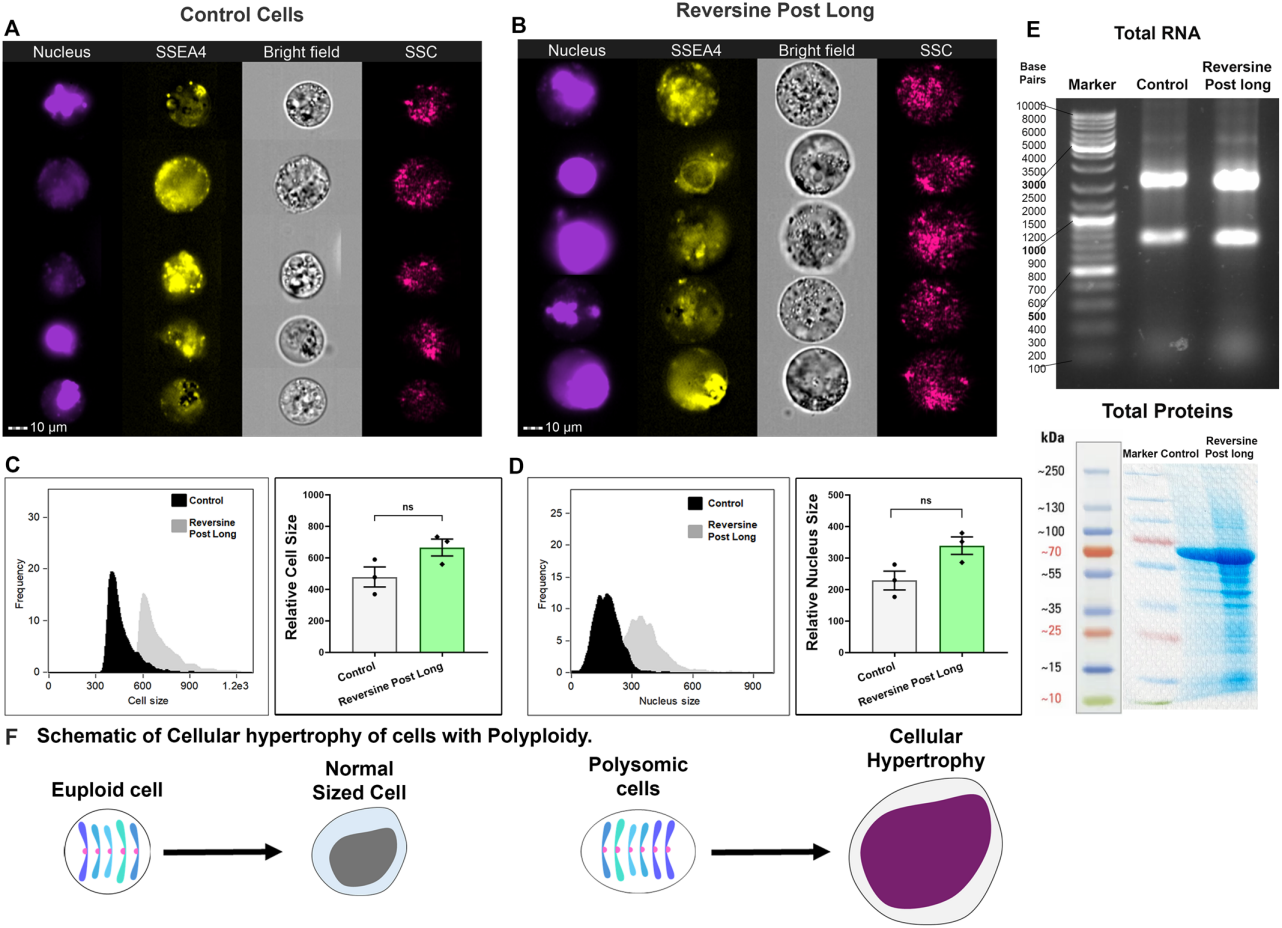
**The surviving iPSCs harbouring supernumerary chromosomes had increased cell size with higher cellular RNA and protein content.** (A) Representative cell images of control cells obtained by image flow cytometry depicting normal cell size. Scale bar: 10 µm. (B) Representative cell images of reversine post long cells obtained by image flow cytometry depicting larger cell size. Scale bar: 10 µm. (C) Plot of cell area obtained by image flow cytometry of control and reversine post long cells with quantification of cell size difference. (D) Plot of nucleus area obtained by image flow cytometry of control and reversine post long cells with quantification of nuclear size difference. (E) Quantification of total cellular RNA and proteins by electrophoresis. (F) Schematic representation of results of this section. *n*=3 experimental repeats; paired two-tailed *t*-test. ns, *P*>0.05. Error bars denote mean±s.e.m.

Interestingly, we observed an increase in cell size in the reversine post long iPSC colonies. We confirmed the relative cell size increase by imaging flow cytometry (Fig. 5A,B). The cell areas of reversine post long cells were greater than those of control iPSCs (Fig. 5C). The increased cell size was accompanied by a concomitant increase in nuclear size, confirmed both microscopically and through imaging flow cytometry (Fig. 3A, Fig. 4B and Fig. 5A,D). At this point, it would be safe to assume that iPSCs with supernumerary chromosomes would express RNA transcripts excessively and overproduce proteins in accordance with chromosome copy number. The abundance of gene transcripts and overproduction of proteins in iPSCs would result in increased cell size. To verify this assumption, we extracted whole RNA and proteins from the reversine post long cells and semi-quantified them by electrophoresis. Indeed, we observed increased production of both RNA and proteins in reversine post long cells compared to control cells (Fig. 5E). A schematic representation of the results of this section can be found in Fig. 5F.

## DISCUSSION

Aneuploidy is a major cause of pregnancy losses. Aneuploidies are generally incompatible with somatic cell growth and physiology and, by extension, proper development of most mammalian tissues, with exceptions of certain tissues adult and extraembryonic tissues. Though aneuploid cells are frequently detected in the developing human embryo, the consequences of aneuploidy on embryonic cellular physiology remain elusive. This study aimed at unravelling some cellular responses to aneuploidy induction in pluripotent stem cells, which could be extrapolated to epiblast cells in developing embryos.

We successfully induced aneuploidies in iPSCs through reversine treatment. The karyotyping data verified the presence of altered chromosome numbers in the iPSC models. Chromosome numbers varied across cells, indicating the generation of a nonspecific aneuploid pool of iPSCs. We corroborated the karyotyping data with flow cytometry-based cell cycle data. We confirmed the maintenance of pluripotency after acquisition of aneuploidy in the iPSCs by immunocytochemical analysis of pluripotency biomarkers OCT4, NANOG and SSEA4, and found that aneuploid iPSCs retain pluripotent characteristic despite their discrepant chromosome numbers. This suggests that chromosome copy number variations and the resulting gene dosage imbalances had not interfered with preservation of pluripotency. Whether the pluripotency network is dosage insensitive or sophisticated dosage compensation mechanisms are involved in persistence of pluripotency has yet to be determined.

Embryoid bodies which *in vitro* mimic the formation of three germ layers during gastrulation in embryos were effectively derived from the iPSCs with altered chromosomes. qPCR was used to establish that the embryoids expressed biomarkers of the three germ layers, suggesting that the fundamental trait of differentiation potential was not lost in aneuploid iPSCs. These findings are in agreement with *in vivo* studies reporting development of tetraploid (4n) embryos up to mid-gestation before onset of detrimental development. (Zhang et al., 2016; Frade et al., 2019).

The aneuploid iPSCs displayed typical DNA damage marks and responded with exaggerated apoptosis. They possessed lagging chromosomes as visible in the anaphase and their prophase nuclei had accumulated a high micronuclei content, both of which denote DNA damage in response to chromosome mis-segregation. The visible DNA damaged response accompanied upregulation of p53 and culminated in excess apoptosis as assessed by qPCR and protein activity assays of caspase 3.

Interestingly, by inducing high levels of chromosome mis-segregation followed by continuous culturing for more than ten passages, we generated iPSCs with supernumerary chromosomes with complex karyotypes. These polyploid iPSCs are stable and actively proliferate despite the presence of whole-chromosome imbalances, without any signs of DNA damage in the form of micronuclei or cell death responses of apoptosis. However, they have increased cell size and volume. These data highlight the previous research showing that pluripotent cells have high tolerance for aneuploidies (Mayshar et al., 2010; Ben-David et al., 2014; Deng et al., 2023).

To summarise, we have shown that whole chromosome aneuploidies can be chemically induced in iPSCs. Due to the chromosomal imbalances, the cells displayed signs of DNA damage, cellular stress and cell death. We also showed, for the first time, that chemically induced aneuploid iPSCs with supernumerary chromosomes can be stably propagated *in vitro* for further studies. Overall, this study provides noteworthy insights pertaining to possible outcomes of chromosomally altered pluripotent cells within the embryonic epiblast.

## MATERIALS AND METHODS

### Cell culture

The human iPSC line SORMi002-A, which has been previously validated (Shivaraj and Parveen, 2019), was used in this study. This cell line has been authenticated and tested for contamination. iPSCs were maintained in conventional pluripotency medium consisting of KnockOut™ DMEM (#10829018), 20% KnockOut™ Serum Replacement (#10828028), 20 mM Glutamax (#35050061), 1× MEM Non-Essential Amino Acids (#11140050), 100 units/ml Penicillin and 100 μg/ml Streptomycin (#15640055) and 5.5 mM 2-Mercaptoethanol (#21985023), all from Gibco™, ThermoFisher Scientific, plus 8 ng/ml bFGF (Sigma-Aldrich, #F0291), on Mitomycin C (Sigma-Aldrich, #M0503) inactivated mouse embryonic fibroblast feeders (iMEFs). The iPSCs were passaged by dissociating the colonies with StemPro™ Accutase™ Cell Dissociation Reagent (Gibco™, ThermoFisher Scientific, #A1110501) and replating on iMEFs in pluripotency medium containing small-molecule Y-27632, a Rho Kinase Inhibitor (Sigma-Aldrich, #Y0573) to prevent single-cell dissociation-induced apoptosis (Watanabe et al., 2007). Medium changes were every alternate day.

### MTT assay

MTT (HIMEDIA^®^, #206-069-5) assay was performed to evaluate iPSC viability upon treatment with different concentrations of reversine. Equally seeded iPSCs were incubated for 48 h with 0.25 µM, 0.5 µM, 0.75 µM and 1.0 µM reversine. After treatment, cells were incubated with 12 mM MTT for 4 h at 37°C. After incubation, the generated formazan was solubilised with DMSO and incubated for an additional 10 min at 37°C. The absorbance was read at 570 nm using an EnSight™ Multimode Plate Reader (Perkin Elmer), and absorbance values were plotted to interpret the optimal concentration of reversine for use in further experiments.

### Chromosomally altered cell generation

For induction of chromosome alterations, iPSCs were treated with 0.5 µM reversine (Sigma-Aldrich, #21985023) for 24 and 48 h. After the treatment durations, medium containing reversine was replaced with fresh pluripotency medium without reversine and processed for further experiments. After removal of reversine, a batch of reversine 48 h iPSCs was maintained in culture for 60 days and then used for further experiments.

### Karyotyping

Giemsa staining and karyotyping experiments were outsourced to Aster Labs. A routine Giemsa staining and karyotyping protocol was followed to obtain and analyse chromosome spreads.

### Cell cycle analysis

Hypotonic propidium iodide staining solution [50 µg/ml propidium iodide (Sigma-Aldrich, #P4170), 0.1% trisodium citrate dihydrate (HIMEDIA^®^, #GRM1415), 0.3 µl/ml Nonidet P-40 (Sigma-Aldrich, #56741) in distilled water] was added to iPSCs and scraped using a cell scraper to harvest the nuclei. The harvested suspension was centrifuged at 850 ***g*** for 5 min. The obtained pellet was resuspended and incubated in 1 ml staining solution. The nuclei were analysed using a BD LSR II flow cytometer (BD Biosciences) with BD FACSDiva™ software (BD Biosciences), and the data were processed using FCS Express 6 software (https://denovosoftware.com/). Per experimental condition, 10,000 events were recorded.

### Embryoid body generation

To generate embryoid bodies, iPSCs from all treatment groups were aggregated in Petri dishes with pluripotency medium without bFGF for 8 days. On day 8, the RNA was extracted from embryoid bodies and converted to cDNA. The gene expression profile of spontaneously differentiated ectoderm, mesoderm and endoderm cells was analysed by qPCR using gene-specific primers.

### Immunocytochemistry

The cells were fixed with 4% paraformaldehyde (Sigma-Aldrich, #P6148) for 30 min. The cells were permeabilised with 0.3% Triton X-100 (HIMEDIA^®^, #MB031) for 15 min, followed by blocking with 3% BSA (Sigma-Aldrich, #A7030) for 30 min, and then incubation with primary antibodies overnight, with the secondary antibodies for 5 h and with DAPI solution for 1 min. Each step was followed by three 5-min washes. All steps were done at room temperature. The 4% paraformaldehyde was prepared in phosphate buffered saline (Sigma-Aldrich, #P4417). Permeabilising buffer, blocking buffer, antibody diluent and DAPI solution were prepared in PBST [PBS containing 0.05% Tween-20 (HIMEDIA^®^, #MB067)]. All antibodies were diluted in 1% BSA. Images were captured under a Nikon Eclipse TE2000 epifluorescence microscope attached to a QCapture camera. Antibodies are listed in Table S1.

### RNA isolation and gene expression analysis by qPCR

RNA samples were isolated from iPSCs and embryoid bodies using TRIzol reagent (Invitrogen™, #15596026). Then, 5 µg RNA was reverse transcribed to cDNA using SuperScript™ IV First-Strand Synthesis System (Invitrogen™, #18091050) according to the manufacturer’s protocol. qPCR for specific genes was performed using TB Green^®^ Premix Ex Taq™ (Takara, #RR420). Primer sets used in the study are listed in Table S2. Cycle threshold (Ct) values were obtained using default settings for the QuantStudio 5 Design and Analysis Software v1.5.2. The endogenous expression of beta-actin was used as an internal control to normalise the expression of target genes. The relative fold change in gene expression was calculated using delta-delta Ct method.

Total RNA from 6×10^5^ cells of each of the treatment groups was extracted for the quantification experiments. Total RNA samples were electrophoresed on 2% agarose gels in 1× Tris Acetate EDTA buffer to quantify the RNA content of iPSCs.

### Caspase 3 assay

To quantify apoptosis, caspase 3 activity was detected using the ApoAlert™ Caspase-3 Fluorescent Assay Kit (Takara, # 630215), which provides a substrate for caspase 3. The assay was carried out as per the manufacturer's instructions. Briefly, the protease activity was measured as a shift in fluorescence from 400 nm to 505 nm due to accumulation of fluorescent product, 7-amino-4-trifluoromethyl coumarin (AFC), due to caspase 3 activity. A standard calibration curve was prepared by plotting respective fluorescence intensities versus 4 µM, 2 µM, 1 µM and 0.5 µM concentrations of AFC. Caspase activity was calculated using the standard calibration plot. All fluorescence measurements were recorded using the EnSight™ Multimode Plate Reader with a 400-nm excitation filter and 505-nm emission filter.

### Protein quantification

The proteins were isolated from an equal number (6×10^5^) of iPSCs of different treatment groups using RIPA buffer (Sigma-Aldrich, #R0278) supplemented with protease inhibitor cocktail (Sigma-Aldrich, #P8340). Then, 10 µl of each protein sample was diluted with 6× SDS-PAGE loading buffer and incubated at 98°C for 5 min. The samples were electrophoresed on a precast Tris-Glycine mini gels (Invitrogen™, #XP04200BOX). A prestained protein ladder (ThermoFisher Scientific™, #26619) was used as molecular marker. Following electrophoresis, the resolved proteins were stained with Coomassie Brilliant Blue R-250. After destaining, the gels were imaged using a Bio-Rad ChemiDoc XRS+Gel Imaging system and processed using Image Lab™ software (Bio-Rad).

### Image flow cytometry

iPSCs from all treatment groups were dissociated into a single-cell suspension using accutase and resuspended in 2% foetal bovine serum (Gibco™, #10100147) in PBS. The iPSCs were stained with anti-SSEA4 antibody (1:250 dilution) for 3 h. Excess primary antibody was removed by centrifugal washing, and the iPSCs were stained with Alexa Fluor^®^ 555 goat anti-mouse antibody (1:250 dilution) for 3 h at room temperature. Excess antibody was removed by centrifugal washing. This was followed by staining of nuclei with Hoechst 33342 (Invitrogen™, #H3570) for 3 min and removal of excess by centrifugal washes. All staining was performed at room temperature. Cell and nuclear size analysis was performed using an Amnis^®^ ImageStream^®^X Mk II Imaging Flow Cytometer with INSPIRE software. The data were processed using IDEAS 6.2 analysis software. For each condition of biological triplicates, 15,000 events were collected.

### Statistical analysis

Statistical significance of the MTT assay cell viability between control and different treatment conditions was determined using one-way analysis of variance (ANOVA) in GraphPad Prism software. The differences between control and different treatment conditions for three experimental repeats of cell cycle analysis, micronuclei count, qPCR, caspase 3 activity assay, cell size and nucleus size were statistically compared using Student's *t*-test in GraphPad Prism software. Error bars denote mean±s.e.m. The following symbols were used to represent the statistical significance: *P<0.05, **P<0.01, ***P<0.001; ns, *P*>0.05.

## Supplementary Material

10.1242/biolopen.061814_sup1 Supplementary information
