# Supplementary material for: Chromosome number alterations cause apoptosis and cellular hypertrophy in induced pluripotent stem cell models of embryonic epiblast cells
Source: Biol Open. 2025 Jan 24;14(1):BIO061814. doi: 10.1242/bio.061814 (PMC11789280; doi:10.1242/bio.061814)
Supplement: Supplementary information [file biolopen-14-061814-s1.pdf]

Table S1. Antibodies used in the study

Antibody	Manufacturer and Catalogue number	Dilution
Anti Oct3/4	BD Pharmingen™, 561555	1:300
Anti Nanog	Cell Signalling Technology, D73G4	1:300
Anti SSEA-4	BD Pharmingen™, 560073	1:300
Alexa Fluor™ 488 Goat anti-Rabbit Antibody	Invitrogen, A-11008	1:500
Alexa Fluor™ 555 Goat anti-Mouse Antibody	Invitrogen, A-21422	1:500

Table S2. Primer sets used in the study

Target gene	5′ Forward sequence 3′	5′ Reverse sequence 3′
BETA ACTIN	TCACCCACACTGTGCCCATCTACGA	CAGCGGAACCGCTCATTGCCAATGG
OCT4	CTGCTGAAGCAGAAGAGGATCAC	CTTCTGGCGCCGGTTACAGAACCA
SOX2	CCCCCGGCGGCAATAGCA	TCGGCGCCGGGGAGATACAT
NANOG	CCTCCTCCATGGTCTGCTTATTCA	CAGGTCTTCACCTGTTTGTAGCTGAG
SOX17	ACATGAAGGTGAAGGGCGAG	TTGTAGTTGGGGTGGTCCTGC
GATA4	TCCAAACCAGAAAACGGAAG	CTGTGCCCGTAGTGAGATGA
PAX6	ATGAACAGTCAGCCAATGGG	CACACCAGGGGAAATGAGTC
P53	CCCTCTCAGTCAGGAAACATTTTC	AGTGGATGGTGGTACAGTCAGAGC
CDKN2A	CAACGCACCGAATAGTTACGGTC	TCTATGCGGGCATGGTTACTG
CASPASE3	CTCGGTCTGGTACAGATGTCGATG	GGTTAACCCGGGTAAGAATGTGCA

Biology Open • Supplementary information
